# Synchrony strategies of six gall inducers that share a superhost, *Eugenia copacabanensis* (Myrtaceae)

**DOI:** 10.1111/plb.70119

**Published:** 2025-10-05

**Authors:** L. P. Nobrega, R. R. Marquesine, P. H. P. Gonçalves, V. C. Maia, D. C. Oliveira, B. G. Ferreira

**Affiliations:** ^1^ Departamento de Botânica, Instituto de Biologia Universidade Federal do Rio de Janeiro, Centro de Ciências da Saúde, Cidade Universitária Rio de Janeiro Brazil; ^2^ Programa de Pós‐Graduação em Ciências Biológicas (Botânica), Departamento de Botânica, Museu Nacional Universidade Federal do Rio de Janeiro Rio de Janeiro Brazil; ^3^ Instituto de Biologia, Programa de Pós‐Graduação em Ecologia, Conservação e Biodiversidade Universidade Federal de Uberlândia Uberlândia Brazil; ^4^ Departamento de Entomologia, Museu Nacional Universidade Federal do Rio de Janeiro Rio de Janeiro Brazil

**Keywords:** Galls, phenology, Restinga, seasonality

## Abstract

- The life cycles of galling insects are synchronized with their host plant phenology, allowing them to better explore available resources. Distinct strategies among six galling species that share a superhost, *Eugenia copacabanensis*, were expected: optimizing growth of galls in times of increased availability of water and other resources.
- The vegetative and reproductive phenology of *E. copacabanensis* in Brazilian Restingas, climate patterns, and development cycles of six gall morphotypes were assessed throughout a year.
- The vegetative phenophases of *E. copacabanensis* presented peaks associated with the rainy season. Reproductive phenophases occurred at two different times: flower buds and flowers in anthesis occurred only during the rainy season, whereas fruits developed and matured during the dry season. Distinct strategies occur among Cecidomyiidae galls. Spiral globoid and clavate galls induced by *Stephomyia* spp. (Cecidomyiidae) are univoltine and occur only at the beginning of the rainy season, with emergence of leaves. Precipitation peaks influenced emergence of leaves and distinct periods of emergence of globoid, leaf‐rolling, and lenticular galls. Multivoltine life cycles occur for globoid and leaf‐rolling galls induced by *Dasineura* spp., lenticular Cecidomyiidae galls, and fusiform Hymenoptera galls.
- The different life cycles among the gall inducers allow exploitation of plant nutritional resources at different times and oviposition sites, thereby avoiding potential nutritional competition. Thus, gall inducers of *E. copacabanensis* exhibit strategic adjustments that enable them to occupy the same host and ensure completion of their life cycles.

## Graphical abstract

Galling insects on the superhost *Eugenia copacabanensis* have developed different life cycle strategies to synchronize with plant seasonal changes and avoid competition for resources.

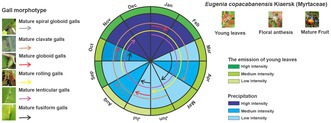

## INTRODUCTION

The life cycle of galling insects depends on availability of reactive sites in their host plants for gall induction and development (Pfeffer *et al*. 2018), which is the most efficient strategy for exploring host resources (Oliveira *et al*. 2016). Reactive sites for gall induction include meristematic tissues, which can be altered by stimuli from galling organisms, and living plant cells of already differentiated tissues, such as parenchymatic tissues, that are capable of re‐differentiation under stimuli from gall inducers, even when galls are induced in mature organs (Oliveira & Isaias 2010; Isaias *et al*. 2025). The longest availability of reactive sites for gall induction, such as during leaf flushing, occurs when there is high availability of water and nutrients for host plants (Weis *et al*. 1988; Carneiro *et al*. 2013; Oliveira *et al*. 2013). Thus, abiotic factors, such as temperature, precipitation, seasonality, water availability, and occurrence of natural fires, can influence phenology of host plants and, consequently, alter availability of sites for gall inducers (Oliveira *et al*. 2013; Rezende *et al*. 2019; Marquesine *et al*. 2024). In cases of asynchrony between gall induction periods and the availability of host organs on plants, the quality and quantity of food resources available to gall inducers may be affected (Yukawa 2000; Oliveira *et al*. 2016). Therefore, synchronization between the developmental cycle of the gall inducer and phenology of the host plant ensures high specificity of galls (Oliveira *et al*. 2013).

Co‐occurring species of gall inducers on a single plant species – a superhost (*sensu* Isaias *et al*. 2013) – may adopt different strategies during their life cycles, ensuring greater availability of induction sites and reduced competition for resources. The life cycles of gall‐inducing species may also exhibit asynchrony among themselves, but remain synchronized with availability of sites of induction and resources of host organs on their host plants (Oliveira *et al*. 2013; Costa *et al*. 2022). Hence, fully expanded leaves may also serve as reactive sites for some gall inducers (Oliveira & Isaias 2009; Nobrega *et al*. 2021). Another potential strategy for distinct galling species sharing the same host organ is to exploit different induction microsites, occupying spatially segregated niches (Inbar *et al*. 2004; Oliveira *et al*. 2013). Furthermore, synchronization between the development cycle of gall inducers and phenology of superhosts is also influenced by competitive pressure from other associated guilds, such as parasitoids and inquilines (Oliveira *et al*. 2016; Rezende *et al*. 2023).

The life cycle of gall‐inducing insects can be univoltine or multivoltine, depending on adaptations to the abiotic and biotic variables that affect plants and their associated galls; in other words, the number of generations each species produces in a year may vary (Yukawa 2000). In subtropical and tropical species, different strategies have been observed. For example, galls induced on *Schinus polygama* (Cav.) Cabrera (Anacardiaceae) by *Calophya mammifex* Burckhardt & Basset (Calophyidae), have a univoltine cycle (Guedes *et al*. 2018a), while galls induced on *Psidium myrtoides* O. Berg. (Myrtaceae) by a triozid (Psylloidea) have a bivoltine cycle (two annual generations) (Carneiro *et al*. 2013), and different species of *Lopesia* (Cecidomyiidae) on *Mimosa gemmulata* Barneby (Fabaceae) exhibit a multivoltine cycle (Costa *et al*. 2018). Moreover, structurally more complex and nutritionally richer galls have been associated with gall inducers that have longer development cycles within these structures, as found in a comparative study of galls induced by different *Lopesia* spp. on *M. gemmulata* (Costa *et al*. 2021).

Superhost plants are commonly found in Restinga environments and are likely to harbour gall‐inducing species and galls with diverse strategies to optimize exploitation of host resources, as precipitation in these environments occurs in nearly every month of the year (Benevides *et al*. 2015; Oliveira *et al*. 2022; Rodarte *et al*. 2022), without the remarkable seasonality that occurs in the Cerrado and Caatinga environments. Restingas are sandy plains with open herbaceous and shrub vegetation and are found in post‐beach regions and on dunes. They are associated with Atlantic forests and occur along the Brazilian coastline (Scarano 2002; Costa *et al*. 2022). The plants that grow in this environment have high ecological importance, as they protect and stabilize sandy soils and allow natural drainage (Melo‐Junior & Boeger 2015; Costa *et al*. 2022), which also contributes to the preservation of diverse gall‐inducing insects (Maia 2018). In these environments, galls create a hydrated and protected microenvironment, offering suitable conditions for completing their life cycle compared with those of free‐living animals (Price *et al*. 1987; Fernandes & Price 1992; Stone & Schönrogge 2003).

As a potential model for studying the influence of abiotic factors on the phenology of associated superhost plants, we selected the superhost *Eugenia copacabanensis* Kiaersk (Myrtaceae), which harbours at least eight leaf gall morphotypes and two bud galls (Maia 1993a, 1993b, 2001, 2019; Maia *et al*. 2002; Monteiro *et al*. 2004; Carvalho‐Fernandes *et al*. 2016). *Eugenia copacabanensis* is an evergreen shrub, reaching up to 6 m in height, and has stems with an outer bark that is shed in sheets, glabrous, elliptical and coriaceous leaves, obovate flower buds, and pear‐shaped, yellow to orange fruits (Souza & Morim 2008; Martins *et al*. 2021). To assess the synchrony of the development cycles of different gall inducers in *E. copacabanensis*, we examined: (1) whether precipitation peaks influence on leaf flushing peaks; (2) whether the life cycle of gall‐inducing insects is influenced by leaf flushing phenophases but without complete overlap; and (3) whether phylogenetically related gall inducers induce galls during the same period of the year.

## MATERIAL AND METHODS

### Study area

The phenological analyses of *E. copacabanensis* were performed in a population located in Restinga da Barra de Maricá (RBM), part of the Maricá Environmental Protection Area (APA‐Maricá), municipality of Maricá, Metropolitan Region of Rio de Janeiro, Brazil, with authorization granted by the Instituto Estadual do Ambiente (INEA; number 063/2022). The Restingas are coastal sandy plains formed from sediments of Quaternary origin, and their flora originates from Atlantic Forests. They are characterized by sandy soils with low water retention, low air humidity, and strong sea wind action (Scarano 2002). Although seasons are not well defined in the RBM, the months of July and August are characterized by a water deficit (Rodarte *et al*. 2022). A total of 15 *E. copacabanensis* individuals distributed in the RBM (22°57′37″S, 42°51′51″W; Fig. S1) were marked with sequentially numbered yellow plastic cable ties and monitored.

**Fig. 1:**
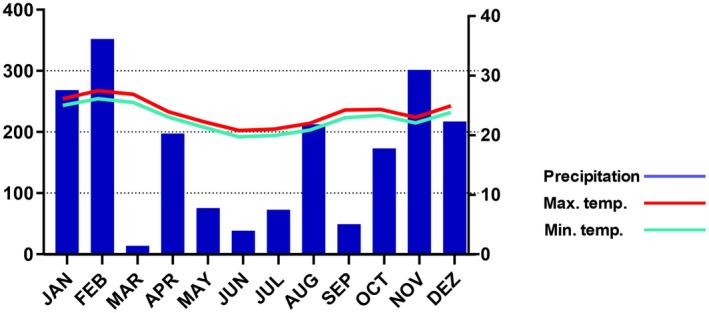
Climogram of Restinga da Barra de Maricá from 2018 to 2023. Red line indicates average maximum monthly temperature (°C), green line indicates average minimum monthly temperature (°C), and blue bars indicate monthly precipitation (mm). Data were obtained through INMET (2018–2023) from an automatic station (A667) in Saquarema, Sampaio Correa, Rio de Janeiro, Brazil.

Meteorological data for the study location were collected from the database of INMET ‐ Instituto Nacional de Meteorologia do Brasil (2024). Data from the automatic station (A667) Saquarema, Sampaio Correia – Rio de Janeiro (22°52′12.0″S, 42°36′36.0″W) were considered for the analysis because of availability of monthly data throughout the phenological study. According to Alvares *et al*. (2013), the climate in Maricá is classified as “AW”, humid tropical, with a hot and rainy summer and a dry winter in the Koeppen classification. The average maximum temperature is 24–27°C, and average minimum temperature is 23–26°C, considering the last 6 years (2018–2023) (Fig. 1). The monthly sums of precipitation during this period indicated that November, February and April had the highest average rates, whereas May, June and July had the lowest precipitation rates (Fig. 1). The dominant winds in the area are northeast trade winds, which are desiccant and constant throughout the year, and southwest trade winds, which are cold and responsible for storms and low temperatures. This area has a high evaporation rate – 124.5 ± 27.6 mm (Mantovani & Iglesias 2008), which is constant throughout most of the year (Louro & Santiago 1984).

### Reproductive and vegetative phenology

**Fig. 2:**
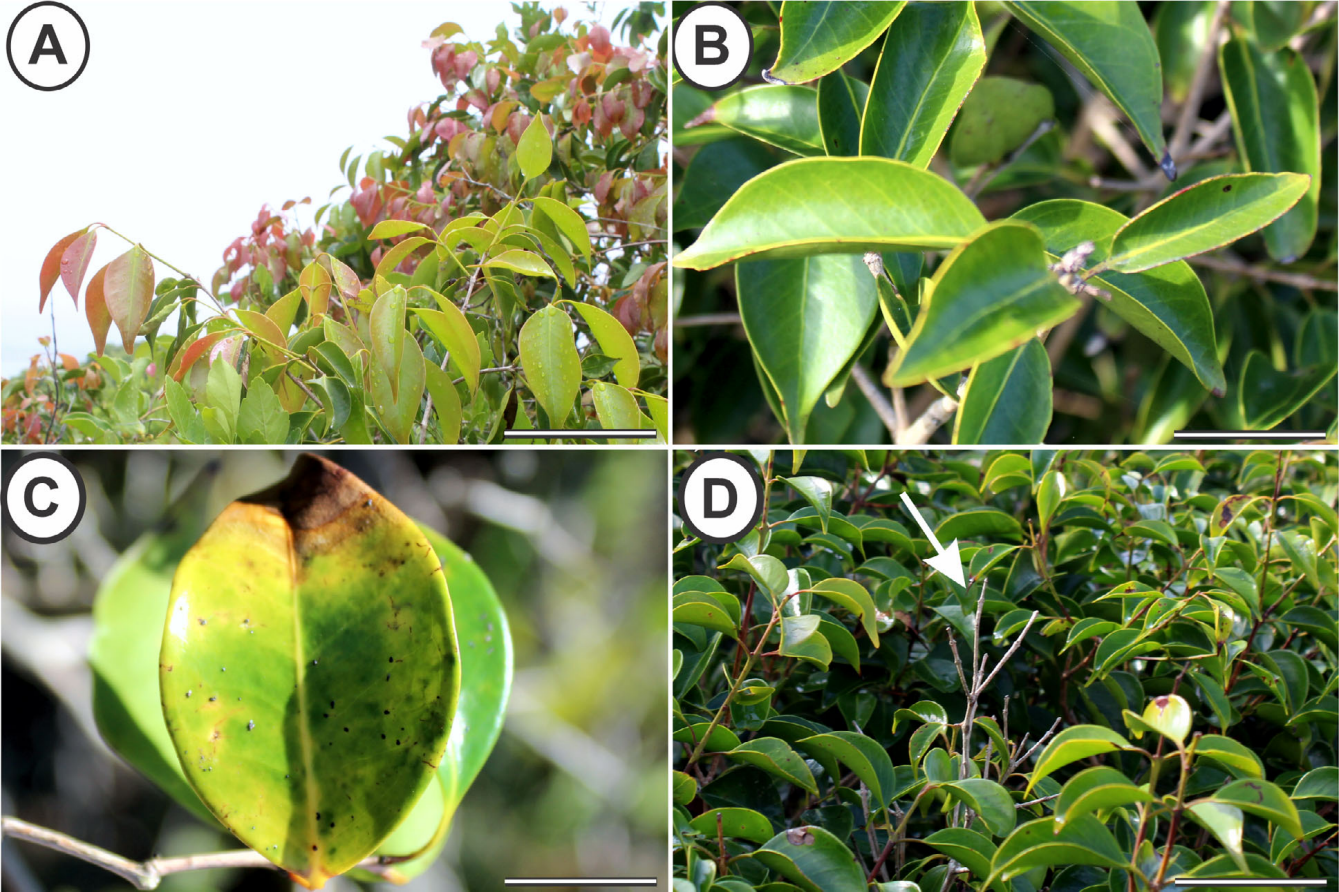
Vegetative phenophases of *Eugenia copacabanensis*. (A) Young leaves; (B) mature leaves; (C) senescent leaves; (D) shoot indicating leaf fall (white arrow). Scale bar: 3 cm.

**Fig. 3:**
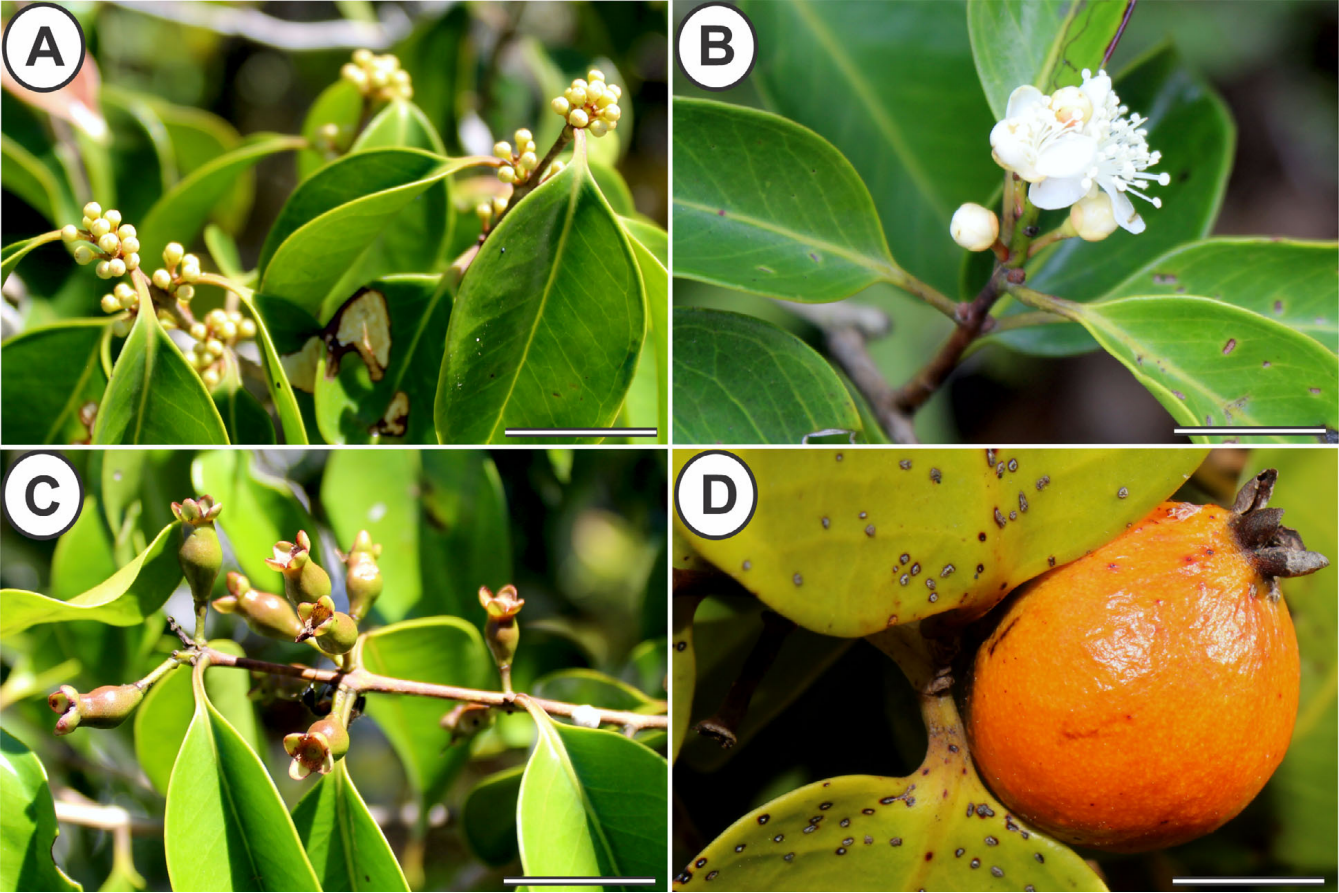
Reproductive phenophase of *Eugenia copacabanensis*. (A) Floral buds; (B) Floral anthesis; (C) Immature fruits; (D) Mature fruits. Scale bar: 3 cm.

Monthly visits were made to 15 selected individuals of *E. copacabanensis* throughout a year (November 2022 to October 2023). The vegetative phenophases (leaf flushing, mature leaves, senescent leaves, and leaf fall; Fig. 2A–D) and reproductive phenophases (young inflorescences, flower anthesis, immature fruits and mature fruits; Fig. 3A–D) were evaluated. The phenophase intensity index was evaluated according to the Fournier method (1974). The Fournier method semi‐quantitatively evaluates the intensity of phenophases in each individual, which, in the present study, was modified into four semi‐quantitative categories (Rodarte *et al*. 2022), in which 0 = absence of phenophase; 1 = 1%–33% canopy cover; 2 = 34%–66%; and 3 = 67%–100%. The intensity indices of Fournier (IF) in each month were expressed using the following formula:

$$\%\;\text{intensity}=\left(\sum \;\text{Fournier}/3\times N\right)\times 100)$$

where ∑ Fournier is the sum of the IF of all individuals; 3 is the maximum value of the semiquantitative categories; and *N* is the total number of plants analysed (Fournier 1974, adapted).

To compare the phenological data obtained, maximum and minimum monthly temperature and precipitation data between the months of November 2022 and October 2023, obtained from the INMET platform (2024), were considered.

### Gall morphotypes characterization and development cycles

The following morphotypes associated with *E. copacabanensis* were studied (Table 1): spiral globoid leaf galls (SGs) induced by *Stephomyia espiralis* Maia, 1993 (Diptera, Cecidomyiidae); clavate leaf galls (CGs) induced by *Stephomyia tetralobae* Maia, 1993 (Diptera, Cecidomyiidae); globoid bud galls (GGs) induced by *Dasineura copacabanensis* Maia, 1993 (Diptera, Cecidomyiidae); young leaf roll galls (RGs) induced by an undescribed species of *Dasineura* (Cecidomyiidae); lenticular leaf galls (LGs) induced by an undescribed species of Cecidomyiidae; and midrib fusiform leaf galls (FGs), induced by an undescribed species in the order Hymenoptera.

**Table 1:** Gall morphotypes induced in *Eugenia copacabanensis* (Myrtaceae).

morphotype	acronym	inducer	chambers per gall	larvae per gall	open/closed
Spiral globoid leaf galls	SGs	*Stephomyia espiralis*	1	1	Closed
Clavate leaf galls	CGs	*Stephomyia tetralobae*	1	1	Closed
Globoid bud galls	GGs	*Dasineura copacabanensis*	1	1	Closed
Young leaf roll galls	RGs	*Dasineura* sp.	1	>10	Open
Lenticular leaf galls	LGs	Cecidomyiidae	1	1	Closed
Midrib fusiform leaf galls	FGs	Hymenoptera	1	1	Closed

The gall morphotypes were described morphologically following Isaias *et al*. (2013), and their development phases were differentiated according to characteristics such as size, shape, colour, and presence of signs of senescence or inducer exit. The gall development was categorized into phases of (1) growth and development, (2) maturation, and (3) senescence according to Rohfritsch (1992) and Arduin and Kraus (1995). The different stages of gall development in *E. copacabanensis* were monitored each month in conjunction with the phenological study of the host plants. The gall phases were also evaluated semi‐quantitatively according to the methods of Fournier (1974), with the modifications described above. The activity index, or the percentage of individuals presenting the morphotype at a given stage of development, was also recorded.

### Statistics

To understand how the intensity indices of the vegetative and reproductive phenophases and the different phases of gall induction adjust, pie charts were constructed, and statistical comparisons between leaf flushing intensity indices of the different development stages of the six gall morphotypes, precipitation and the different phenophases of *E. copacabanensis* were performed using the Watson–Wheeler test with Cramér–von Mises distribution in the R software (R Core Team). The Circular package (Agostinelli & Lund 2024) was used to compare circular data with Watson‐Wheeler test, and Plotrix (Lemon *et al*. 2023) was used for plotting the circular visualizations.

## RESULTS

### Characterization of gall morphotypes associated with *E. copacapanensis*

**Fig. 4:**
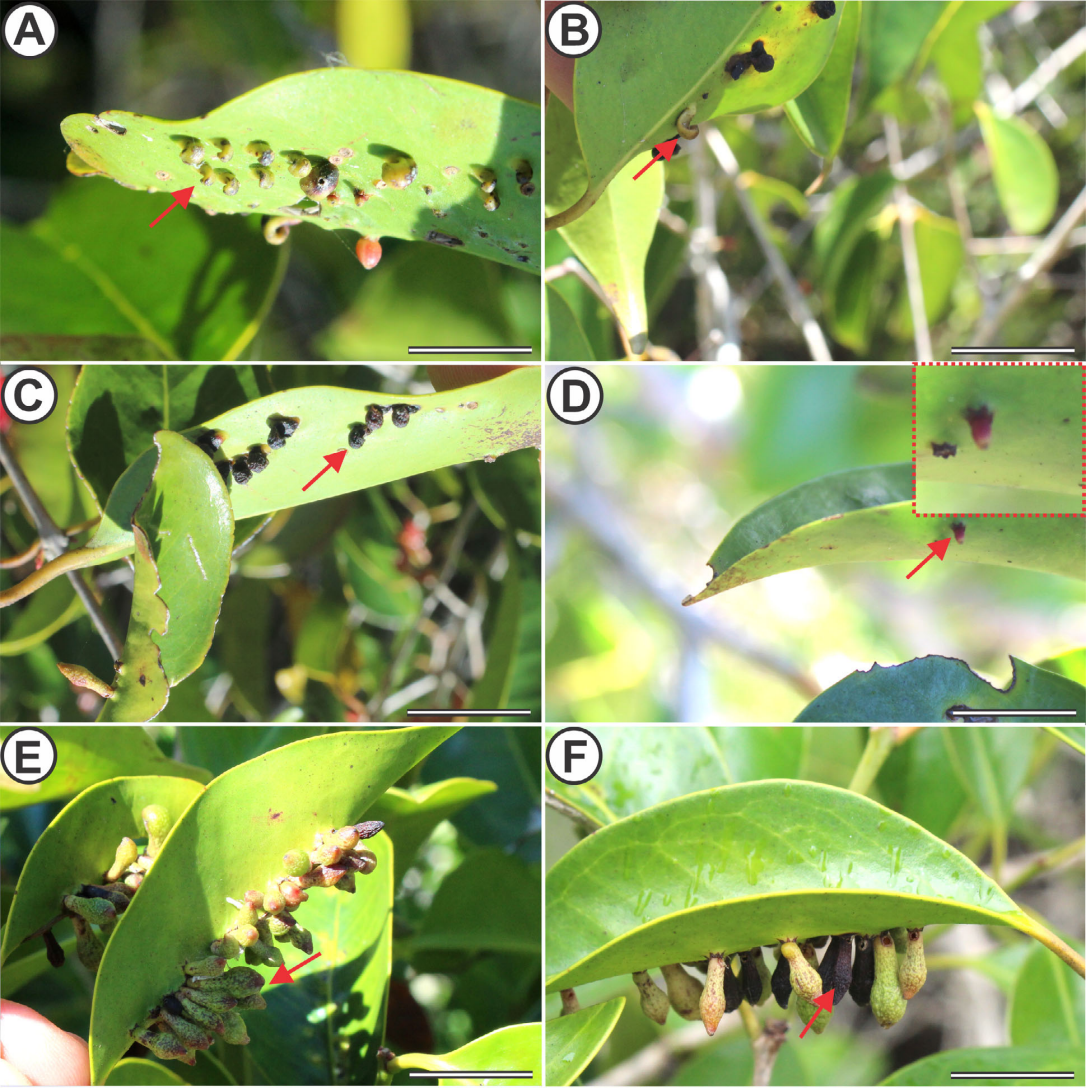
Galls induced by *Stephomyia* spp. on *Eugenia copacabanensis* leaves. (A–C) Spiral globoid galls (SGs) of *S. spiralis*; (A) young galls; (B) mature galls; (C) senescent galls; (D–F) clavate galls (CGs) of *S. tetralobae*; (D) young galls; (E) mature galls; (F) senescent galls. Scale bar: 3 cm.

The spiral globoid galls (SGs) induced by *S. espiralis* are extralaminar and can be induced on both adaxial and abaxial surfaces of *E. copacabanensis* leaves. *S. espiralis* galls are unilocular (have a single gall chamber) containing a single gall‐inducing larva. The young stage (YSG) is light green and slightly folded at the apical portion (Fig. 4A). At maturation stage (MSG), these galls are yellowish green and more tightly coiled, resembling a spiral in lateral view (Fig. 4B). In the senescent stage (SSG), galls are either still green or turn brownish, with the inducer exiting through a hole in the middle of the gall (Fig. 4C).

The clavate galls (CGs) induced by *S. tetralobae* are extralaminar and induced on the abaxial surface of host leaves. With respect to the number of larval chambers, those of *S. tetralobae* are unilocular (one gall chamber), hosting a single gall‐inducing larva. The young stage (YCG) is reddish, an elongated fusiform shape and thin (Fig. 4D). When mature (MCG), galls gradually lose their reddish colouration, presenting brownish spots and a greyish‐green colouration. The apical portion of the MCG is dilated, making the morphotype resemble a club (Fig. 4E). In the senescence stage (SCG), these galls become yellowish or brownish, presenting an exit hole of the insect in the basal portion in contact with the non‐galled portion of the leaf (Fig. 4F).

**Fig. 5:**
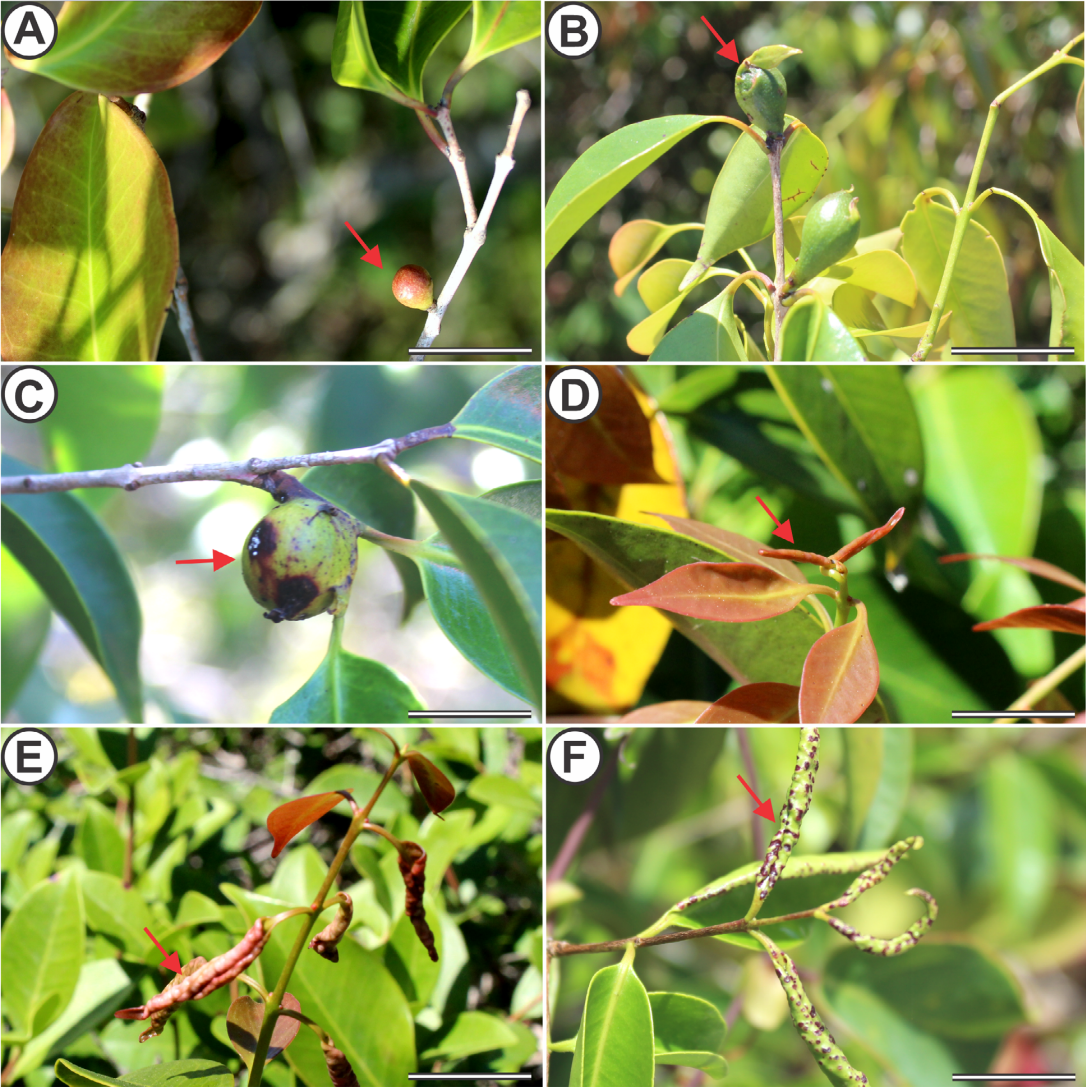
Galls induced by *Dasineura* spp. on *Eugenia copacabanensis* buds and leaves. (A–C) Globoid bud galls (GGs) of *D. copacabanensis*; (A) young galls; (B) mature galls; (C) senescent galls; (D–F) leaf‐rolling galls (RGs) of *Dasineura* sp. (D) young galls; (E) mature galls; (F) senescent galls. Scale bar: 3 cm.

Globoid bud galls (GGs) are induced by *D. copacabanensis* in lateral buds of *E. copacabanensis* These GGs are unilocular and contain one gall‐inducing larva. When young (YGG), they are small, with average heights of 11.0 mm and diameters of 7.0 mm, and reddish in colour (Fig. 5A). In the mature stage (MGG), these galls are reddish in the apical portion and a dark green in the middle and basal portions (Fig. 5B). In the senescence stage (SGG), the galls become brownish and have an inductor exit orifice in the apical portion (Fig. 5C).

The leaf rolling galls (RGs) induced by *Dasineura* sp. are formed from the complete rolling of young leaves of *E. copacabanensis*. This gall morphotype has an open larval chamber formed by rolling of the young leaf, which is occupied by one or, commonly, a few gall‐inducing larvae. The young stage (YRG) is reddish, similar to the colour of young leaves of the host (Fig. 5D). In the mature stage (MRG), it is reddish to light green, with some reddish spots (Fig. 5E). In the senescence stage (SRG), these galls are green with brownish spots (Fig. 5F). In addition, the rolled leaf becomes more rigid than at other stages.

**Fig. 6:**
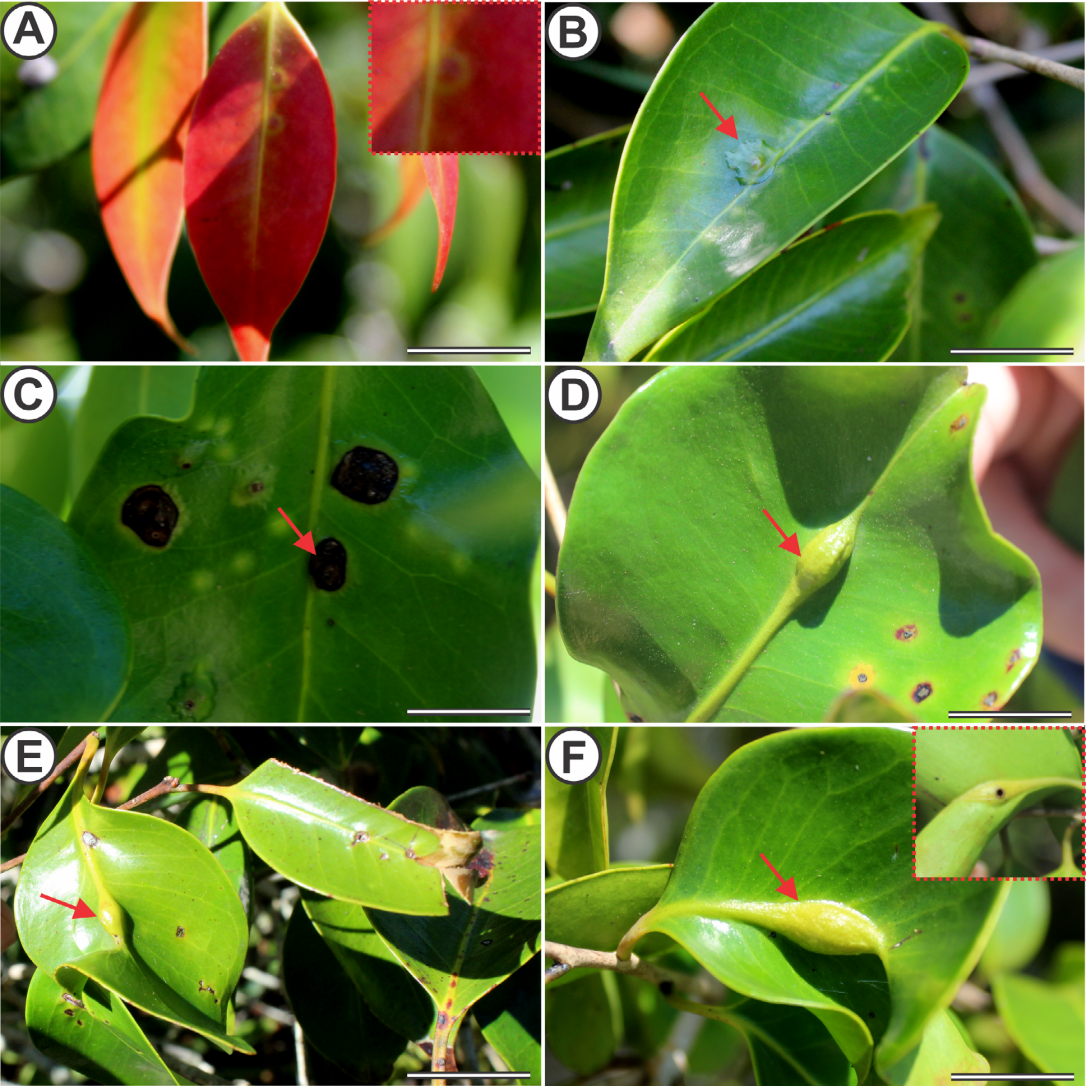
Lenticular Cecidomyiidae galls and fusiform midrib Hymenoptera galls induced on *Eugenia copacabanensis* leaves. (A–C) Lenticular Cecidomyiidae galls (LGs); (A) young galls; (B) mature galls; (C) senescent galls; (D–F) fusiform midrib galls (FGs) induced by Hymenoptera; (D) young galls; (E) mature galls; (F) senescent galls, top right with details of the inductor exit hole. Scale bar: 3 cm.

The lenticular galls (LGs) induced by Cecidomyiidae are intralaminar and induced on the abaxial side of the *E. copacabanensis* leaf. LGs are unilocular, containing one gall‐inducing larva. In the young stage (YLG), they are reddish to yellowish and occur on young leaves (Fig. 6A). The mature stage (MLG) is dark green (Fig. 6B). Senescent galls (SLGs) are light green or sometimes brownish, with the exit orifice on the adaxial or abaxial side of the leaf (Fig. 6C).

The hymenopteran‐induced fusiform galls (FGs) are intralaminar and induced in the midrib of mature leaves of *E. copacabanensis*. FGs are unilocular, containing one gall‐inducing larva. The young (YFG) (Fig. 6D), mature (MFG) (Fig. 6E) and senescent (SFG) stages of these galls are light green and smaller (up to 2.0 mm height and 2.3 mm diameter in young galls). The senescent stage presents an exit hole of the inducer on the abaxial face of the gall (Fig. 6F).

### Climate data

**Fig. 7:**
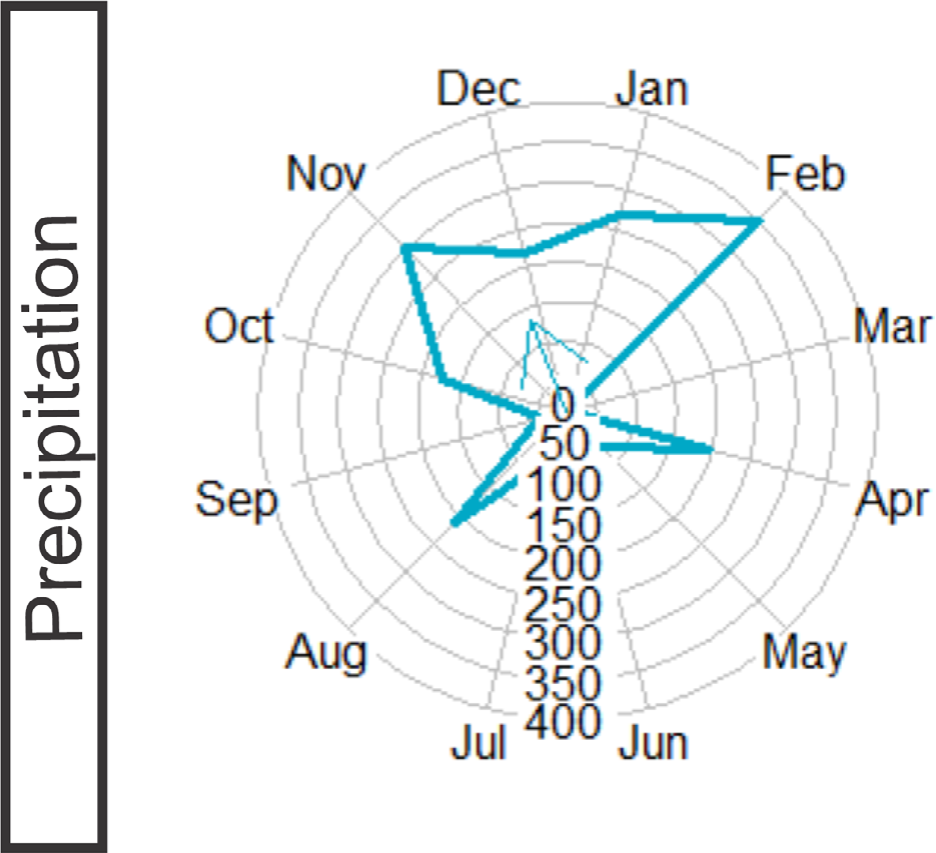
Circular analysis of precipitation rates from November 2022 to October 2023. Solid blue line indicates precipitation during 1 year of phenological study. Values indicate accumulated monthly precipitation in millimetres.

During the monitoring period, there were two major peaks in precipitation: in November 2022 (301.40 mm) and February 2023 (352.00 mm). The average precipitation vector was between November and December (Fig. 7). Average temperature did not significantly vary throughout the year, ranging between 20 and 30°C. According to the climate data, the season of highest rainfall occurs between August and February, whereas the months with lower precipitation occur between March and July 2023, although April showed a peak (Fig. 7).

### Vegetative phenophases

**Fig. 8:**
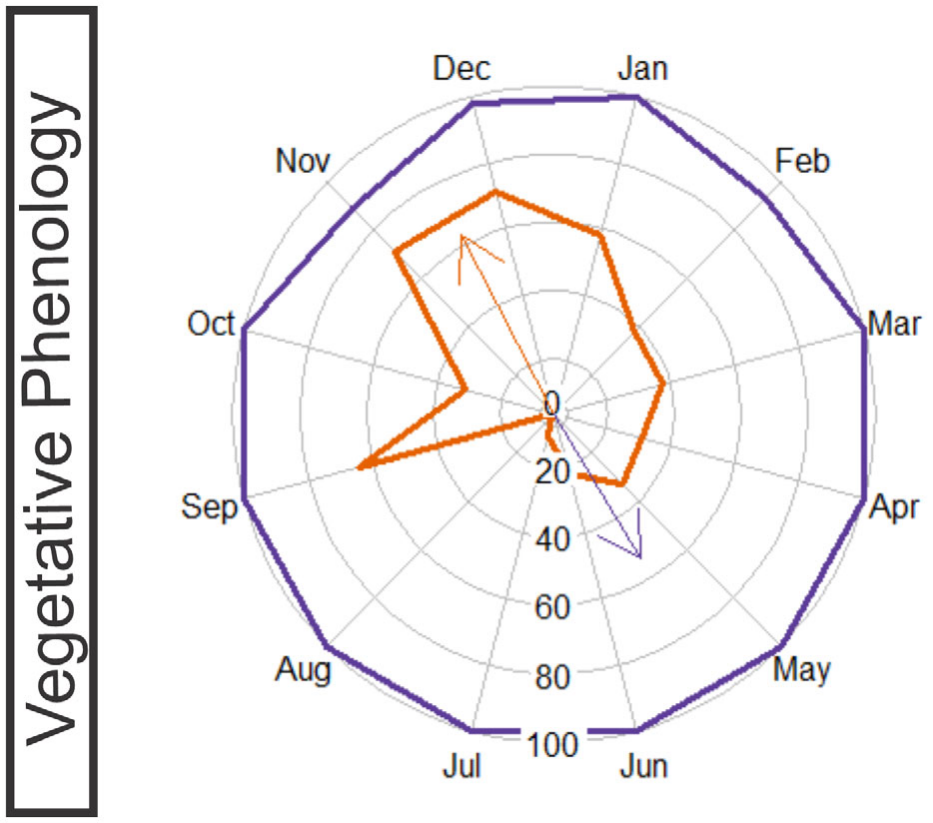
Circular analysis of intensity indices of vegetative phenophases – young and mature leaves – of *Eugenia copacabanensis* from November 2022 to October 2023. Solid orange line: young leaves; solid purple line: mature leaves. Values are percentages of intensity of each phenophase.

The start of leaf flushing (Fig. 2A) of *E. copacabanensis* began in September and continued until July, lasting 10 months. The highest peaks of this phenophase occurred in September (IF = 64.44%), November, and December (IF = 71.10%), with the average vector recorded between November and December (Fig. 8). However, young leaves were observed throughout the entire rainy season, with higher intensity until January and lower intensity between February and March, as well as a peak in May following a peak in precipitation in April. According to the Watson–Wheeler test, there were no significant differences between the circular distributions of the precipitation periods and the occurrence of young leaves (*W* = 5.44; *P* = 0.066). Thus, there is some similarity between the distributions of precipitation and the emergence of young leaves, with some overlap, especially during the rainy season.

Expanded leaves (Fig. 2B) appear consistently throughout the year (IF = 100.00%), with the average vector occurring between May and June (Fig. 8). The monthly precipitation and the occurrence of expanded leaves did not have a statistically similar circular distribution (*W* = 144.30; *P* < 0.001), indicating that these distributions do not overlap.

The leaf senescence phenophase (Fig. 2C) occurred throughout the year, with the highest peak in February (IF = 55.60%) and the average vector in December (Fig. S2A). There was no similarity between distributions of precipitation and leaf senescence (*W* = 31.97; *P* < 0.001), indicating that these distributions do not overlap. The leaf fall phenophase (Fig. 2D) occurred from April to February and lasted 10 months (Fig. 2A). The highest peak of this phenophase occurred in November (IF = 46.67%), with the average vector between September and October. There was no similarity between the distributions of precipitation and leaf drop, meaning that these distributions do not overlap (*W* = 45.94; *P* < 0.001).

### Reproductive phenophases

Floral buds (Fig. 3A) in *E. copacabanensis* presented three low‐intensity peaks throughout the year. The first peak was from November to February, lasting 2 months, the second peak was in April, and the third peak was in June (Fig. S2B). The highest peaks of this phenophase occurred in January (IF = 20.00%), February (IF = 13.33%) and April (IF = 24.40%), with the average vector occurring between January and February. The peaks of floral bud production were more intense during the rainy season, despite an unusually large peak in April, which coincided with a peak in precipitation in this month. There was no similarity between the distributions of precipitation and the occurrence of floral buds, indicating that these distributions do not overlap (*W* = 36.27; *P* < 0.001). The floral anthesis phenophase (Fig. 3B) was recorded in September, December, and February (Fig. S2B). The highest peak of this phenophase occurred in February (IF = 6.70%), and the average vector pointed between December and January. There was similarity between distributions of precipitation and floral anthesis, meaning that these distributions overlapped (*W* = 3.95; *P* = 0.139).

The immature fruit phenophase (Fig. 3C) occurred from March to November and lasted 9 months throughout the year (Fig. S2C). The highest peaks of this phenophase were in March (IF = 15.60%) and May (IF = 17.80%), with the average vector occurring between May and June, following the peaks of floral buds and floral anthesis, and continuing until November. The difference between the periods of precipitation and the availability of immature fruits was statistically significant (*W* = 86.85; *P* < 0.001). Therefore, there is no similarity between the distributions of precipitation and immature fruits, hence these distributions do not overlap.

Mature fruits (Fig. 3D) were recorded from July to August and then from October to November, with each period lasting 1 month, one during the dry season and the other at the start of the rainy season (Fig. S2C). The highest peak occurred in July (IF = 13.33%), with the average vector occurring between July and August. The differences between distributions of the precipitation periods and occurrence of mature fruits were statistically significant (*W* = 27.51; *P* < 0.001). Therefore, there is no similarity in the circular distribution between precipitation‐exposed fruits and mature fruits, hence these distributions do not overlap.

### Development cycle of spiral globoid galls

**Fig. 9:**
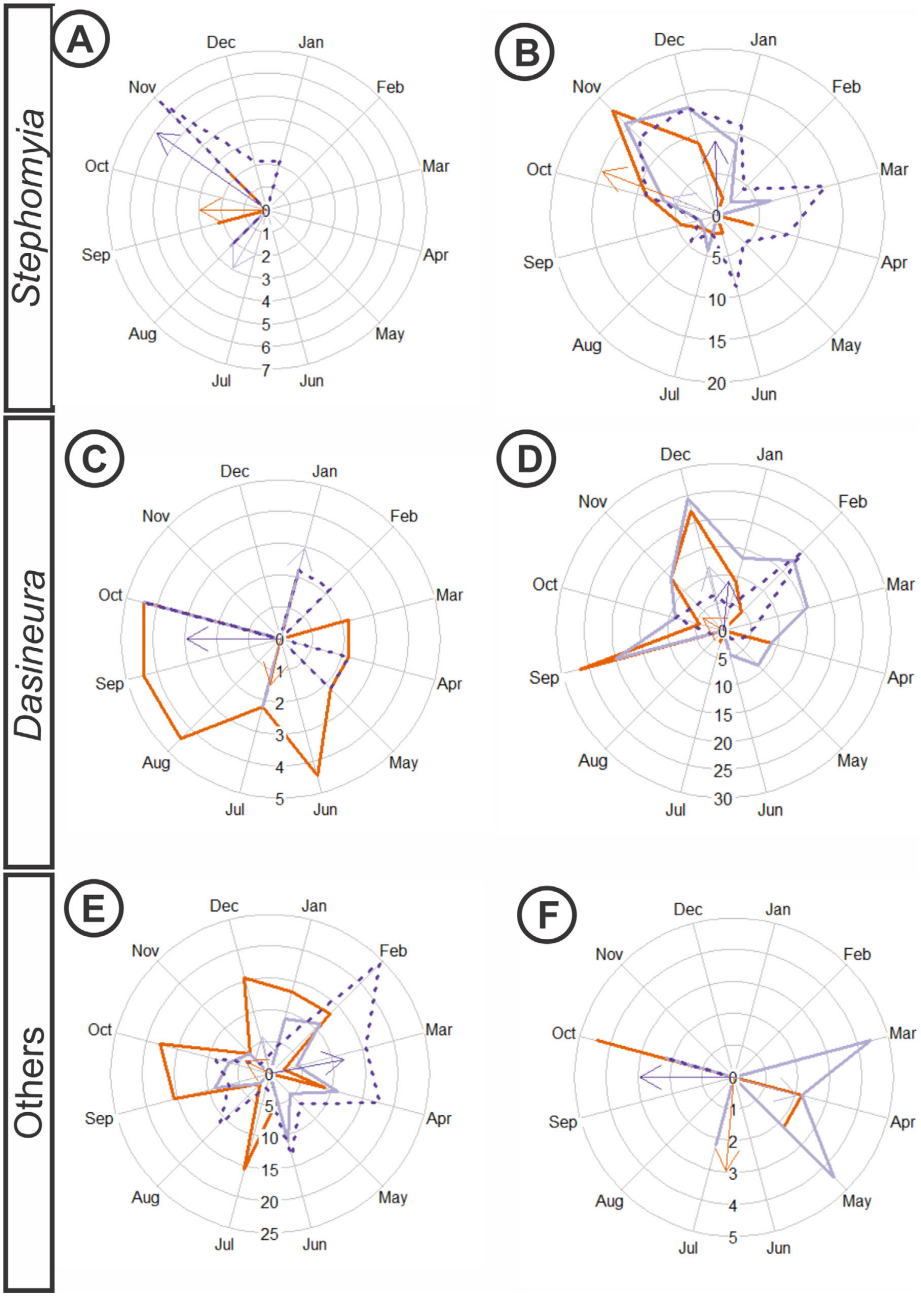
Circular analysis of life cycle intensity index of *Eugenia copacabanensis* galls from November 2022 to October 2023. (A, B) Galls induced by *Stephomyia* spp. (C, D) Galls induced by *Dasineura* spp. (E, F) Lenticular Cecidomyiidae galls (LG) and fusiform Hymenoptera galls (FG). (A) SG development cycle; (B) CG development cycle; (C) GG development cycle; (D) EG development cycle; (E) LG development cycle; (F) FG development cycle. Solid orange line represents young galls; solid lilac line represents mature galls; and dotted purple line represents senescent galls. Values are percentages of intensity of each life cycle.

The appearance of spiral globoid galls (YSGs) induced by *Stephomyia spiralis* on *E. copacabanensis* leaves began in September and continued until November. Peaks occurred in September and November (IF = 2.22%), with the average vector between September and October (Fig. 9A). There were statistically significant differences between young leaves and YSG (*W* = 6.40; *P* = 0.041) and between precipitation and YSG (*W* = 6.84; *P* = 0.033), indicating that these distributions do not overlap.

Mature spiral globoid galls (MSGs) were observed in August, lasting 1 month. The peak occurred in August (IF = 2.22%), with the average vector occurring between July and August. The distributions of young leaves and MSG (*W* = 4.01; *P* = 0.135) and precipitation and MSG (*W* = 4.00; *P* = 0.135) did not differ significantly. Therefore, the occurrence of MSGs is similar to the distributions of young leaves and precipitation.

Senescent spiral globoid galls (SSGs) occurred in August and from November to February, each lasting 1 month. The peaks occurred in August (IF = 2.22%) and November (IF = 6.67%), with the average vector between October and November (Fig. 9A). There was no similarity between the periods of precipitation and the occurrence of senescent galls (*W* = 11.92; *P* = 0.003).

### Development cycle of clavate galls

The appearance of young clavate galls (YCG) induced by *S. tetralobae* on *E. copacabanensis* leaves began in June and continued until January, with an atypical peak in April (Fig. 9B). The highest peaks occurred between October and December, with maximum intensity in November (IF = 17.78%) and the average vector between October and November. The cycles of availability of young leaves and the occurrence of young galls were statistically distinct (*W* = 21.23; *P* < 0.001), as were the cycles of precipitation and the occurrence of YCG (*W* = 26.31; *P* < 0.001).

Mature clavate galls (MCGs) occurred from July to March, lasting 8 months. The peak occurred in November (IF = 15.56%), with the average vector between October and November. The cycles of availability of young leaves and MCG were not similar, meaning that these distributions do not overlap (*W* = 20.56; *P* < 0.001), as did the comparison between MCG and precipitation (*W* = 26.75; *P* < 0.001).

Senescent clavate galls (SCGs) occurred from October to August, lasting 10 months. The peaks occurred in November and December (IF = 13.33%) and March (IF = 13.33%), with the average vector between December and January (Fig. 9B). There were no significant differences between periods of precipitation and occurrence of senescent galls (*W* = 1.24; *P* = 0.538), indicating similarity in distribution of precipitation and occurrence of senescent galls, which overlap.

### Development cycle of globoid galls

Young globoid galls (YGG) induced by *Dasineura copacabanensis* on the lateral buds of *E. copacabanensis* were recorded between January–February and April–May, with each period lasting 1 month. Peaks occurred in January and February (IF = 2.22%), April and May (IF = 2.22%), and October (IF = 4.44%), with the average vector in January. The period with the highest intensity for YGG occurred during the rainy season. The periods of availability of young leaves and the occurrence of YGG did not differ statistically (*W* = 1.93; *P* = 0.380), nor did the periods of precipitation and YGG (*W* = 1.92; *P* = 0.383), suggesting that the cycle of YGG is related to the cycles of young leaves and precipitation.

The number of mature globoid galls (MGGs) peaked in July, October and January. The highest peak was in October (IF = 4.44%), with the average vector occurring between September and October (Fig. 9C). There were no significant differences between availability of young leaves and mature galls (*W* = 2.94; *P* = 0.230) or between MGG and precipitation (*W* = 3.45; *P* = 0.178), indicating similarity in distribution between young leaves and MGG and between precipitation and MGG, indicating that these distributions overlap.

Senescent globoid galls (SGGs) were observed from January to October. Peaks occurred in June (IF = 4.44%), August, September and October (IF = 4.44%), with the average vector between June and July (Fig. 9C). The distribution of SGG differed statistically from that of precipitation (*W* = 21.24; *P* < 0.001), indicating that these distributions do not overlap.

### Development cycle of leaf rolling galls

The appearance of young leaf‐rolling galls (YRG) induced by *Dasineura* sp. on young leaves of *E. copacabanensis* began in September and was recorded until February, lasting 5 months (Fig. 9D). The period of occurrence of YRG was most intense during the rainy season, with a smaller peak in April. Peaks occurred in September (IF = 26.67%) and December (IF = 22.22%), with the average vector between October and November. The distributions of young leaf availability and the occurrence of YRG were significantly different (*W* = 10.09; *P* = 0.006), indicating that these distributions do not overlap, as was the comparison between precipitation and YRG (*W* = 22.98; *P* < 0.001).

Mature rolling galls (MRGs) occurred from September to June (Fig. 9D). The highest intensity peaks were in December (IF = 24.44%) and September (IF = 20.00%), with the average vector between December and January. There were no significant differences between distributions of young leaves and MRGs (*W* = 4.34; *P* = 0.114), indicating similarity in distributions of young leaves and MRGs and that these distributions overlap. The same was true for MRG and precipitation (*W* = 0.25; *P* = 0.881), indicating similarity between distributions of precipitation and mature galls.

Senescent rolling galls (SRGs) occur from September to June. The peak occurred in February (IF = 20.00%), with the average vector between December and January (Fig. 9D). There was no similarity between the distributions of precipitation and senescent galls, indicating that these distributions do not overlap (*W* = 6.96; *P* = 0.031).

### Development cycle of lenticular galls

The appearance of young lenticular galls (YLGs) induced by unidentified Cecidomyiidae on *E. copacabanensis* leaves occurred from June to April, with various peaks throughout the year (Fig. 9E). Peaks occurred in July (IF = 15.56%), September (IF = 15.56%), October (IF = 17.78%) and December (IF = 15.56%), with the average vector between October and November. Four peaks in YLG occurred in both rainy and dry seasons. The period of availability of young leaves was similar to that of young galls (*W* = 4.37; *P* = 0.124). However, there was a significant difference between the period of precipitation and the occurrence of YLG (*W* = 6.75; *P* = 0.034).

Mature lenticular galls (MLGs) occurred from January to November. Peaks were in February (IF = 11.11%), April (IF = 11.11%), June (IF = 11.11%) and September (IF = 8.89%), with the average vector in December. The distribution of MLG did not differ from that of young leaves (*W* = 3.22; *P* = 0.200), nor did the distribution of MLG differ from that of precipitation (*W* = 0.56; *P* = 0.757).

Senescent lenticular galls (SLGs) occurred in peaks in February (IF = 24.44%), March (IF = 15.56%), April (IF = 17.78%) and June (IF = 13.33%), with the average vector between March and April (Fig. 9E). The distributions of SLG and precipitation were significantly different (*W* = 59.72; *P* < 0.001).

### Development cycle of midrib fusiform galls

The appearance of young fusiform galls (YFG) induced by unidentified Hymenoptera on *E. copacabanensis* leaves occurred in April, May, July and October (Fig. 9F). Peaks were observed in April (IF = 2.22%), May (IF = 2.22%), June (IF = 2.22%) and October (IF = 4.44%), with the average vector between June and July. The period of occurrence of YFG was different from that of young leaves (*W* = 9.46; *P* = 0.009), as was the period between YFG and precipitation (*W* = 9.95; *P* = 0.007).

Mature fusiform galls (MFGs) occurred from March to May, in July and in October. Peaks were observed in March (IF = 4.44%) and May (IF = 4.44%), with the average vector in April (Fig. 9F). There was no similarity between MFGs and young leaves (*W* = 13.79; *P* = 0.001) or between MFGs and precipitation (*W* = 16.42; *P* < 0.001).

The peak number of senescent fusiform galls (SFGs) occurred in October (IF = 2.22%), with the average vector between September and October (Fig. 9F). There were no significant differences between the occurrence of precipitation and SFG (*W* = 4.00; *P* = 0.135), indicating similarity in the distributions of precipitation and senescent galls and that these distributions overlap.

## DISCUSSION

The phenophases of *E. copacabanensis* adjusted to variations in water availability, resulting in an increase in leaf sprouting just after precipitation peaks. As expected, gall development cycles do not overlap completely, presenting a temporal sequence of peaks of different morphotypes in the rainy season. In contrast, other gall morphotypes presented several peaks throughout the year. The phylogenetically closely related gall inducers have similar synchronization strategies, even with distinct sites of gall induction and gall shapes. The globoid bud galls induced by *D. copacabanensis* and lenticular galls induced by an unidentified species of Cecidomyiidae present several peaks throughout the year, overlapping with the production of young leaves. On the other hand, the galls induced by *Stephomyia* spp., namely the spiral globoid gall of *S. espiralis* and clavate gall induced by *S. tetraloba*, as well as the leaf rolling gall induced by *Dasineura* sp., occur at the beginning of the rainy season but without overlapping peaks. Unlike strategies observed in the Cecidomyiidae morphotype, the number of fusiform galls induced by Hymenoptera in the midrib (FG) peaked at the end of the rainy season and during the period of lower precipitation. Therefore, different strategies in the life cycle occur among the galling species associated with the superhost *E. copacabanensis*, taking advantage of the periods of increased production of leaves and avoiding the overlap of life cycles among the distinct gall inducers.

### Influence of precipitation peaks on phenological cycle of *E. copacabanensis*

Restingas are environments with low seasonality, which influences the phenology of tree and shrub species (Rodarte *et al*. 2008). Restinga plants have continuous periods of leaf flushing and leaf fall, but with low intensity (Rodarte *et al*. 2022), a pattern observed in *E. copacabanensis*, since both phenophases occur throughout nearly the entire year. According to Morellato *et al*. (2000), leaf flushing of Atlantic Forest plants can occur throughout the year, with the influence of the rainy season on the intensity of young leaf production in some species. Leaf sprouting and continuous leaf fall have also been reported in phenological studies of other Myrtaceae species present in Restingas in southern and southeastern Brazil, such as *Psidium cattleyanum* Sabine, *Myrcia brasiliensis* Kiaersk (Bauer *et al*. 2014) and *Eugenia sellowiana* DC. (Castro *et al*. 2020). In fact, water abundance is one of the seasonal factors that most influences plant phenology (Myers *et al*. 1998; Lemos‐Filho & Mendonça‐Filho 2000), such as the leaf flushing (Pfeffer *et al*. 2018), as observed in the current results for *E. copacabanensis*, with peaks after the months of highest precipitation. The peaks of young leaves in *E. copacabanensis* occurred just after the precipitation peaks, including in atypical months during the dry season, as observed in April 2023. Unlike young leaves, expanded leaves in *E. copacabanensis* occur throughout the year, as is also observed in *E. dysenterica* (Mart.) DC. in the Cerrado biome (Camilo *et al*. 2013) and in the *E. hiemalis* Cambess. in riparian forests (Athayde *et al*. 2009). Leaf fall in *E. copacabanensis* occurred more intensely during peak precipitation periods and less intensely during periods of lower precipitation. Although leaf fall can occur during periods of water deficit as an adaptive mechanism of the species (Castro *et al*. 2020), this was not the case for the species under study.

Considering that the synchrony of the galling insect with its host provides better conditions for its development, the leaf flushing of *E. copacabanensis* during almost the entire year allows these insects to induce their galls more frequently throughout the year, thus resulting in a multivoltine life cycle, even though some gall inducers are univoltine, as in *S. spiralis* and *S. tetralobae*. Some gall inducers with a multivoltine life cycle can induce galls on both young and mature leaves (Oliveira *et al*. 2016), as observed for gall inducers in *Copaifera langsdorffii* (Jacq.) (L.) (Fabaceae) and *Avicennia schaueriana* Stapf & Leechm. ex Moldenke (Acanthaceae) (Oliveira & Isaias 2010; Nobrega *et al*. 2021). This allows them to better explore the spectrum of responsiveness (Weis *et al*. 1988) of parenchyma cells in response to galling stimuli (Oliveira & Isaias 2009; Oliveira *et al*. 2016). Therefore, multivoltism is important for gall inducers that share the same host plant, allowing inducers to induce galls without overlap between cycles, as observed for the nine morphotypes of *C. langdorffii* (Oliveira & Isaias 2010).

Regarding reproductive phenophases, the start of flower bud production occurred after peaks in precipitation, mainly in the rainy season but also after the precipitation peak in April, demonstrating its influence on phenophase. As also observed for *E. sellowiana* (Castro *et al*. 2020), the start of the rainy season influences production of floral buds. Subsequent to this, floral anthesis in *E. copacabanensis* occurred at the time of highest precipitation, although at very low intensity. This result is similar to that reported for *E. punicifolia* (Kunth) DC. (Cunha *et al*. 2016), in which occurrence of flowers during anthesis coincided with periods of highest precipitation but was somewhat distinct from that reported for *E. sellowiana* present in Restingas, which peaks only in January (Castro *et al*. 2020). Castro *et al*. (2020) suggested that the low intensity of flowers in anthesis may be related to abiotic factors, such as wind and precipitation, which can lead to the fall of these structures, since Restinga environments are subjected to winds throughout the year. The simultaneous loss of floral buds and flowers after periods of higher precipitation was consequently concomitant with the occurrence of young leaves in *E. copacabanensis*, as also observed in *Campomanesia adamantium* (Cambess.) O. Berg (Myrtaceae) (Nucci 2016). Another factor that may be related to the emergence of flower buds and flower phenophases in anthesis is the photoperiod, as reported by Rodarte *et al*. (2022) in plants from Restinga de Maricá over 7 years, but without any relationship to precipitation. This is contrary to our results, in which such phenophases occurred during periods of precipitation. Thus, our results and those of the other studies demonstrate strategies for the emergence of floral buds and floral anthesis related to the period of precipitation, emission of young leaves, and daylength.

Following floral anthesis, immature fruits begin to develop, being more intense during periods of lower precipitation, as observed in *E. copacabanensis* congeneric species, *E. hiemalis*, *E. involucrata* DC. and *E. uniflora* L. (Athayde *et al*. 2009), which appear to be related to high temperatures. The fruiting of *E. copacabanensis* was supra‐annual in a study of a population of Restinga in Marambaia, Rio de Janeiro, with intervals of more than 1 year between fruiting events (Zamith & Scarano 2004). In the present study, the fruiting of *E. copacabanensis* in the Restinga of Barra de Maricá occurred from March to November, whereas in the study by Zamith & Scarano (2004), in Marambaia, it occurred from July to December. Fruit maturation in *E. copacabanensis* occurred from July to September, similar to *Mimosa gemmulata* (Fabaceae) (Costa *et al*. 2021), with a fruit maturation strategy at the end of the low‐rainfall period. Both fruits and galls are strong sinks for photoassimilates, and there is direct competition for carbon and nutrients among these two organs in the same plant (Larson & Whitham 1991; Dorchin *et al*. 2006). Phenological adjustments among gall inducers and host plant phenology may prevent competition among reproductive structures and galls, allowing better access to nutrients and energy resources for developing organisms inside galls (Larson & Whitham 1991, 1997; Oliveira *et al*. 2017; Guedes *et al*. 2018a). Therefore, the occurrence of cecidomyiid galls in *E. copacabanensis* in periods distinct from those in developing fruits may be a strategy to optimize development of their galls.

### Relationships among precipitation, young leaf production and the gall cycle

The galls induced by six distinct species on *E. copacabanensis* were asynchronous, as also observed for the five species of gall induced by *Lopesia* spp. in *M. gemmulata*. This may avoid competition for feeding sites and energy resources from the host plant (Costa *et al*. 2021). Among the six morphotypes studied, only the young and mature globoid bud galls induced by *D. copacabanensis* and the young and mature lenticular leaf galls induced by an undescribed Cecidomyiidae were significantly related to peaks of highest availability of young leaves, which occurred at distinct times throughout the year. This may be related to a direct relationship between bud activation in growth seasons and leaf flushing, that could influence induction of both bud and leaf galls (Reale *et al*. 2016). For morphotypes in the mature phase, the spiral globoid galls induced by *S. espiralis* and the leaf roll galls induced by *Dasineura* sp. were similar to the availability of young leaves, demonstrating the relationship between the induction of these galls and favourable moments for growth. The relationship between the occurrence of young galls and leaf flushing is a promising strategy for reproductive success of an inducer (Hodkinson 2009), as observed in *Calophya rubra* (Blanchard) (Hemiptera: Psylloidea) galls on *Schinus polygamus* (Cav.) Cabrera (Anacardiaceae) (Guedes *et al*. 2018b), and in some morphotypes of galls induced in the superhost *Copaifera langsdorffii* (Oliveira *et al*. 2013).

Our results indicate that the development cycles of the six gall morphotypes studied differ in number of generations throughout the year and seem to be related to the phylogenetic relationships of the gall inducers. The gall inducers *S. espiralis* and *S. tetraloba* have a univoltine cycle, as observed previously for other Neotropical galling insects (Butignol and Pedrosa‐Macêdo, 2003; Fagundes & Gonçalves 2005; Oliveira *et al*. 2013). The other gall inducers of GG, RG, LG and FG had a multivoltine cycle, which is common in galling cecidomyids of Neotropical regions (Hawkins & Gagne 1989). This multivoltine life cycle is associated with perennial plants and adjusted to environmental conditions and resource availability that allow such behaviour (Carneiro *et al*. 2013; Guedes *et al*. 2018a). Our results confirm these observations, since the GGs, RGs, LGs and FGs induced in *E. copacabanensis* present cycles that are somewhat related to peaks in precipitation and production of young leaves, although in distinct periods of the year.

During periods of precipitation, there is increased emergence and development of young leaves (Santos‐Mendonça & Almeida‐Cortez 2007; Pfeffer *et al*. 2018). Since young leaves are more responsive to galling insect stimuli (Weis *et al*. 1988), there might be increased gall induction during periods of greater young leaf availability (Rohfritsch 1992). Thus, the timing of oviposition tends to be synchronized with phenology of the host plant when more resources are available for gall development (Yukawa 2000; Yukawa & Akimoto 2006).

Some galls, such as in SG and CG induced by species of the genus *Stephomyia*, occurred close to production of young leaves at the beginning of the rainy season. Although they did not overlap completely, peaks of leaf flushing in *E. copacabanensis* occurred in months immediately following peaks in precipitation, demonstrating that production of *Stephomyia* galls can be related to start of the most favourable season, rather than other periods of higher precipitation. Similar behaviour was observed in galls of the lenticular bivalve morphotype of *Lopesia* sp. in *M. gemmulata* in the Caatinga biome (Costa *et al*. 2021).

Lenticular leaf gall inducers have a multivoltine life cycle associated with the continuous leaf flushing, with several peaks throughout the year. Similar strategies occurred in bud galls induced by *D. copacabanensis*, which occur in months of young leaf production, as shown statistically, and peak occurrence of flower buds. Thus, several precipitation peaks throughout the year influence not only the occurrence of young leaves and flower buds, but also the occurrence of bud galls. In contrast, the young leaf‐rolling galls induced by *Dasineura* sp. peaked after leaf flushing but only in a typical rainy season, which differentiates this morphotype from the GGs induced by congeneric species. This difference may be related to protection of a closed gall, such as GG, in periods of desiccation, unlike open leaf‐rolling galls (Butignol and Pedrosa‐Macedo, 2003).

A third strategy was observed for Hymenoptera‐induced fusiform galls on midribs, which appeared mainly at the end of the rainy season, while the mature galls appeared during the dry season. The cycle began at times of lowest precipitation, and senescence occurred during the period of highest precipitation. Thus, these galls may continue growing while remaining protected during the dry season. This strategy is not often reported for other galling species in tropical regions (Barbosa & Fernandes 2019). One example is the gall‐inducing Eriococcids, which may have a diapause in secondary galls induced on phellogen on stems of the same host to overcome the period of leaf fall (Gonçalves *et al*. 2009).

## CONCLUSIONS

Our results revealed three distinct strategies for synchronization of galling with the host: gall induction at the start of a typical rainy season, gall induction synchronized with highest leaf flushing, and gall induction at the end of the rainy season for survival during the dry season. Furthermore, congeneric *Stephomyia* species use a similar strategy, inducing galls only at the beginning of the rainy season, whereas both *Dasineura* spp. had multivoltine cycles related to leaf flushing, as well as lenticular leaf gall, with an unidentified inducer. The midrib galling Hymenoptera had a distinct life cycle: inducing galls at the end of the rainy season and inhabiting the gall during lowest precipitation, a strategy for resistance to environmental drought. Thus, the six gall inducers in *E. copacabanensis* adopt different strategies in association with the host phenological cycle to better explore available resources.

## AUTHOR CONTRIBUTIONS

LPN: Conceptualization; data curation; investigation; methodology; visualization; writing – original draft preparation; writing – review and editing. RRM: Formal analysis; data curation; methodology; writing – original draft preparation; writing – review and editing. PHPG: Formal analysis; writing – original draft preparation; Writing – Review and Editing. VCM: Conceptualization; methodology; validation; writing – review and editing. DCO: Conceptualization; methodology; validation; writing – review and editing. BGF: Conceptualization; investigation; methodology; writing – original draft preparation; writing – review and editing; funding acquisition; project administration; supervision.

## Supporting information


**Fig. S1.** Map of Restinga Barra de Maricá, in the Maricá Environmental Protection Area (APA‐Maricá), Maricá, RJ State, Brazil. Limits of the APA‐Maricá are highlighted in yellow and sampling area is represented by a black dot.
**Fig. S2.** Circular analysis of intensity index of vegetative phenophase –senescent leaves and leaf fall– and reproductive phenology of *Eugenia copacabanensis* from November 2022 to October 2023. (A) Senescent leaf intensity index (solid orange line) and leaf fall intensity index (solid purple line). (B) Flower bud intensity index (solid orange line) and floral intensity index (solid purple line); (C) immature fruit intensity index (solid orange line) and ripe fruit intensity index (solid purple line). Values indicate percentage of intensity of each phenophase.
