# Supplementary material for: Synchrony strategies of six gall inducers that share a superhost, Eugenia copacabanensis (Myrtaceae)
Source: Plant Biol (Stuttg). 2025 Oct 5;28(1):225–39. doi: 10.1111/plb.70119 (PMC12710831; doi:10.1111/plb.70119)
Supplement: Supplementary file 1 — Fig. S1. Map of Restinga Barra de Maricá, in the Maricá Environmental Protection Area (APA‐Maricá), Maricá, RJ State, Brazil. Limits of the APA‐Maricá are highlighted in yellow and sampling area is represented by a black dot. Fig. S2. Circular analysis of intensity index of vegetative phenophase –senescent leaves and leaf fall– and reproductive phenology of Eugenia copacabanensis from November 2022 to October 2023. (A) Senescent leaf intensity index (solid orange line) and leaf fall intensity index (solid purple line). (B) Flower bud intensity index (solid orange line) and floral intensity index (solid purple line); (C) immature fruit intensity index (solid orange line) and ripe fruit intensity index (solid purple line). Values indicate percentage of intensity of each phenophase. [file PLB-28-225-s001.docx]

**Synchrony strategies of six gall inducers that share a superhost, *Eugenia copacabanensis* (Myrtaceae)**

Leticia Ponticel Nobrega^1,2^; Rayssa Rosa Marquesine^1,2^; Pedro Henrique Pereira Gonçalves^4^; Valéria Cid Maia³; Denis Coelho Oliveira^4^; Bruno Garcia Ferreira^1,2^

^1^ Departamento de Botânica, Instituto de Biologia, Universidade Federal do Rio de Janeiro, Centro de Ciências da Saúde, Cidade Universitária, Rio de Janeiro, RJ, 21941-902, Brazil

^2^ Programa de Pós-Graduação em Ciências Biológicas (Botânica), Museu Nacional, Universidade Federal do Rio de Janeiro, Rio de Janeiro, Brazil

^3^ Programa de Pós-Graduação em Zoologia, Museu Nacional, Universidade Federal do Rio de Janeiro, Rio de Janeiro, Brazil

^4^ Universidade Federal de Uberlândia, Instituto de Biologia, Programa de Pós-graduação em Ecologia, Conservação e Biodiversidade, Uberlândia, Brazil

*Corresponding author: Bruno Garcia Ferreira (bgf@biologia.ufrj.br)

**Supporting information**

**
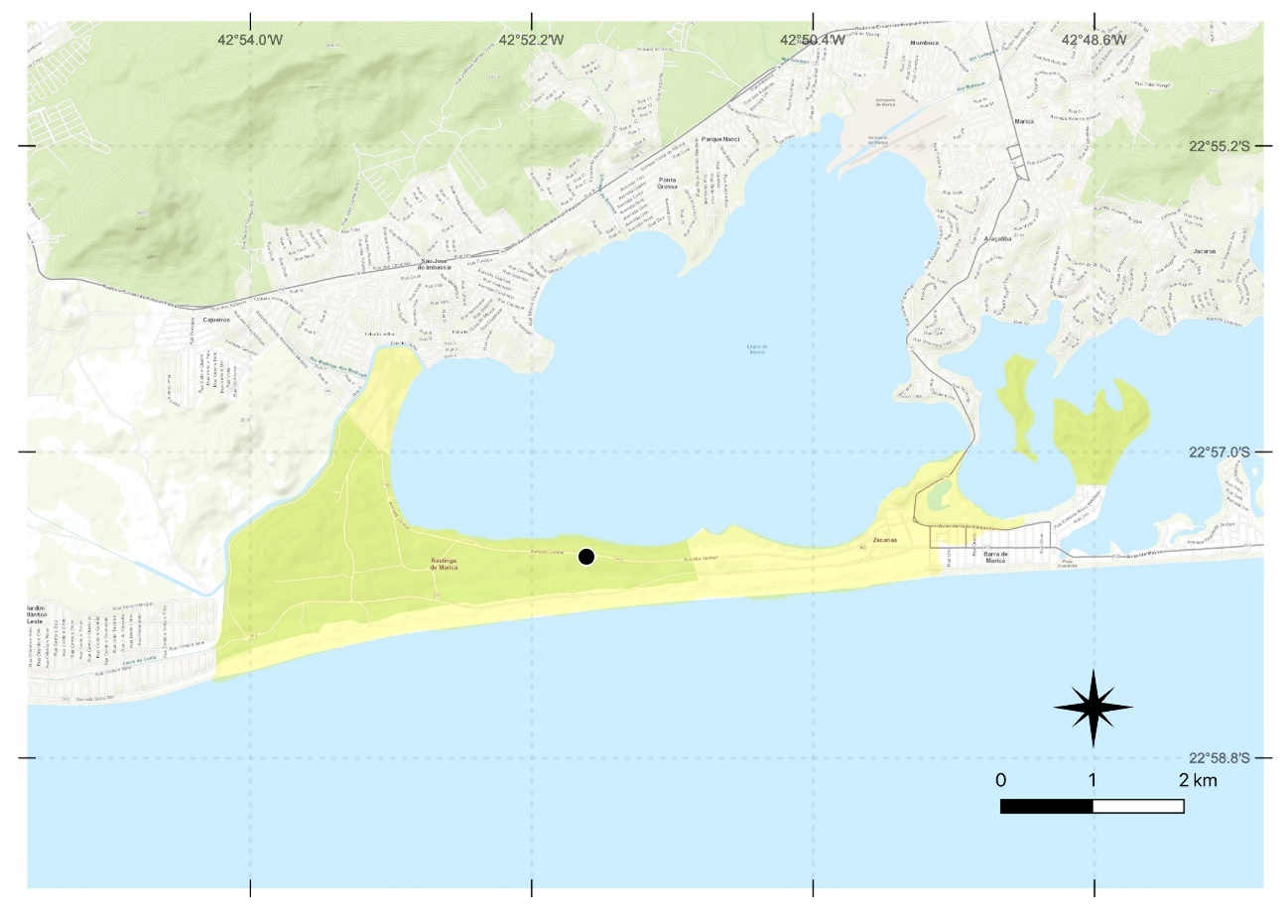
**

**Supplementary Figure 1.** **Map of the Maricá Environmental Protection Area (APA-Maricá), Maricá, RJ State, Brazil**. The limits of the APA-Maricá are highlighted in yellow and the sampling area is represented by the black dot.


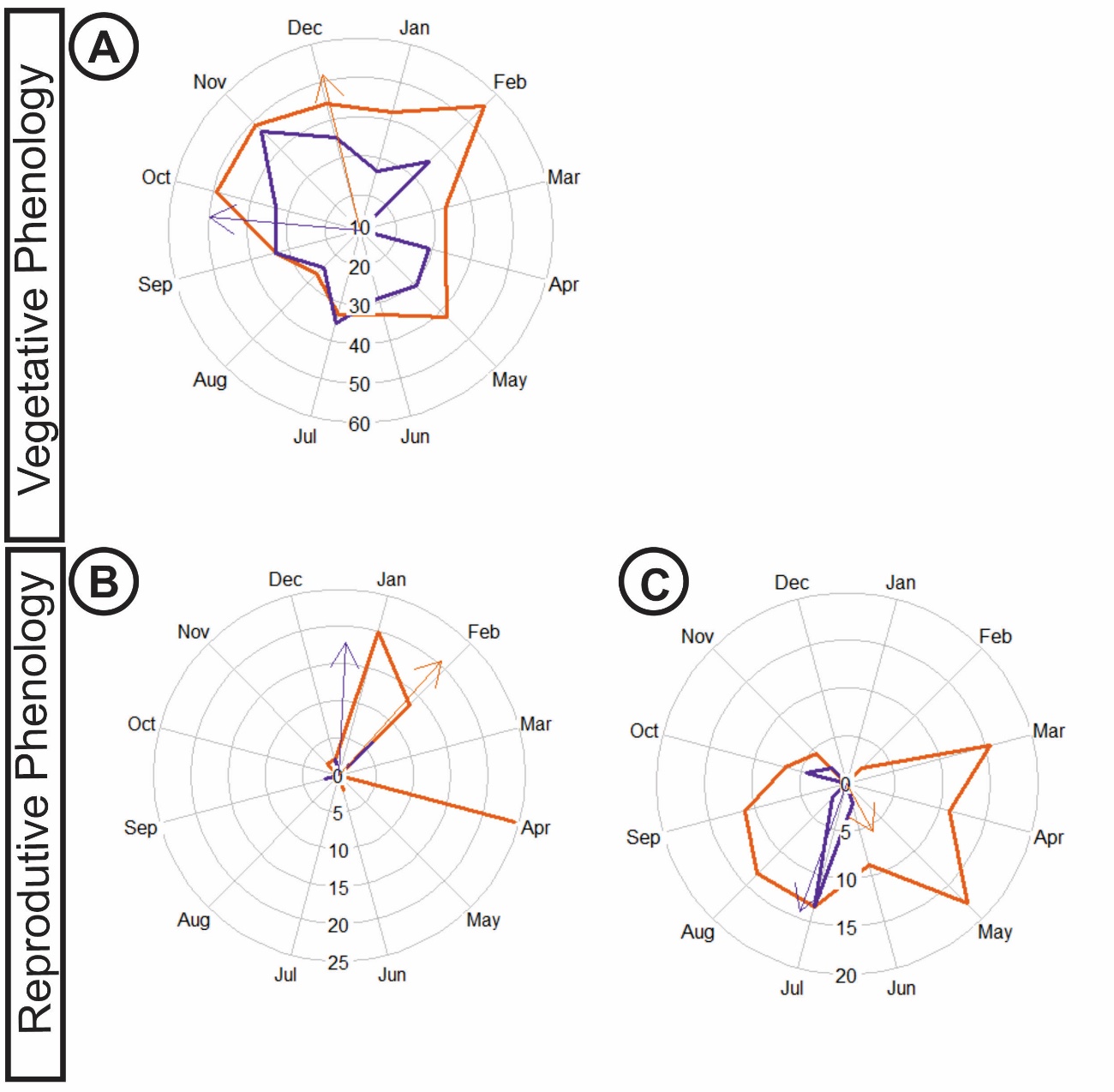


**Supplementary Figure 2.** **Circular analysis of the intensity index of the vegetative phenophase –senescent leaves and leaf fall– and the reproductive phenology of *E. copacabanensis* from November 2022 to October 2023.** **A)** Senescent leaf intensity index (solid orange line) and leaf fall intensity index (solid purple line). **B)** Flower bud intensity index (Solid orange line) and floral intensity index (Solid purple line); **C)** immature fruit intensity index (Solid orange line) and ripe fruit intensity index (Solid purple line). The values indicate the percentage of the intensity of each phenophase.
